# A multidisciplinary approach for rehabilitation following ocular trauma

**DOI:** 10.4103/2321-3868.126093

**Published:** 2014-01-26

**Authors:** Pradeep Kumar, Himanshi Aggarwal, Varun Baslas, Raghuwar Dayal Singh

**Affiliations:** 1 Department of Prosthodontics, Faculty of Dental Sciences, King George’s Medical University, Lucknow, Uttar Pradesh India; 2 King George’s Medical University, Room No. 404, E block, Gautam Buddha Hostel, Lucknow, 226 003 Uttar Pradesh India

**Keywords:** Custom ocular prosthesis, ocular trauma, prosthesis replacement

## Abstract

Ocular trauma is a very common incidence that occurs in up to 67% of patients with maxillofacial trauma. It results in life-long agony of not being like others with two eyes, which can see and admire the nature’s beauty. This article reports on a case of a 23-year-old male patient with phthisis bulbi, resulting from ocular trauma. The patient was rehabilitated aesthetically by fabrication of custom-made ocular prosthesis for his traumatically injured right eye. The patient was pleased with the aesthetic outcome, comfort, and mobility offered by the custom ocular prosthesis. There were no complications with regard to health of underlying residual ocular tissues and there was no need of relining of the prosthesis at 6 month recall appointment. Rehabilitation of patients with ocular trauma requires a multidisciplinary approach involving ophthalmologist, psychologist, and skilled maxillofacial prosthodontist. Custom-made ocular prosthesis fitted over the phthisical globe seems to be a highly positive, logical, noninvasive, and beneficial approach to increase mobility to the prosthesis, improve the cosmetic appearance and psychological well-being of the patient.

## Introduction

An unfortunate loss or absence of an eye due to congenital defect or an acquired defect such as trauma, tumor, and painful blind eye, and so on,[1] causes aesthetic disfigurement of face and also significantly affects the individual physical, psychological, emotional, and social well being.[2] Intraocular injuries occur in up to 67% of patients with maxillofacial trauma.[3] Wound healing secondary to severe trauma may result in an ocular condition known as phthisis bulbi. Phthisis bulbi is not a specific clinical entity; rather, it represents an ocular end-stage disease of various causes and is defined by atrophy, shrinkage, and disorganization of the eyeball and intraocular contents[4] leading to aesthetic disfigurement of face.

**Table 1:** 

Access this article online	
**Quick Response Code:**	**Website:** www.burnstrauma.com
	**DOI:** 10.4103/2321-3868.126093

The pitiable condition of such a patient who is sans eyes is beyond imagination. The patient is not only devoid of a very essential sense organ, but also loses his ability to admire the nature’s beauty. At the same time, he loses the beauty and the charm of his face, as eyes are generally the first facial feature to be noticed and most of the times, when we meet other people, eyes speak earlier than the words.[5] Majority of patients experience extreme stress due to functional disability and nonacceptance in the society, thereby deteriorating the patient’s quality of life. Replacement of the lost eye as soon as possible is necessary to promote physical and psychological healing for the patient and to improve social acceptance.[6]

Rehabilitating such patients requires a multidisciplinary approach involving the combined and timely efforts of an ophthalmologist, psychologist, and a skilled maxillofacial prosthodontist.[7] Once it is established by the ophthalmologist that there is no possibility of preserving the patient’s vision, usually the sensitive and painful injured eye is enucleated followed by fitting of stock eye. But it should be emphasized that the custom ocular prosthesis can be fitted over the phthisical eye as contrary to the usually employed practice of enucleating the injured eye. Ocular prosthesis fitted over the phthisical globe and customized according to the patient’s socket tissue bed and individualized aesthetic requirements is a highly positive, logical, noninvasive, and beneficial approach to improve the cosmetic appearance and psychological well-being of the patient.

## Case report

**Figure 1:**
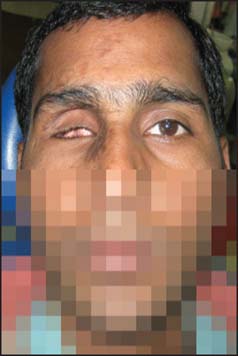
Preoperative photograph showing defect of right eye.

A 23-year-old male patient reported to the department of prosthodontics with the chief complaint of loss of his right eye sight completely and unaesthetic appearance due to regressing eye ball [Figure 1]. There was a history of trauma 3 years back, followed by gradual loss of eyesight of right eye. He consulted several ophthalmologists but could not regain the eyesight. Patient was referred to the department of ophthalmology, where the condition was diagnosed as “phthisis bulbi”. Visual-evoked potential tests and slit lamp examination confirmed that there was no possibility of regaining vision of right eye.

Patient had a very low self-confidence and his interviewing gave indication of psychological distress, as a result of his appearance. So, before beginning with the replacement procedure for his lost eye, psychological consultation sessions along with anxiolytic drugs were administered. Group meetings with other patients who had been successfully rehabilitated were also arranged to allay the patient’s anxiety.

A careful examination of the defect area revealed shrunken and non-inflammed healthy Grade 0 socket[8] with adequate depth of fornices. On palpation it was found that, there was no associated pain, discomfort, or residual edema. So, it was planned to fabricate custom ocular prosthesis for the patient. Whole procedure was explained to the patient/guardian to gain his cooperation. The procedure was initiated by fabricating a custom impression tray, using autopolymerizing polymethyl methacrylate (PMMA) resin (Trevalon, Dentsply India Pvt. Ltd., Gurgaon, India) in its dough stage. A needle-less syringe was attached to the center of the impression tray to serve as a handle. The tray was checked in the patient’s eye socket and adjusted. Before the impression was made, the tray was perforated to aid the retention of impression material and to allow escape of excess material that could compress and distort the ocular tissues.

**Figure 2:**
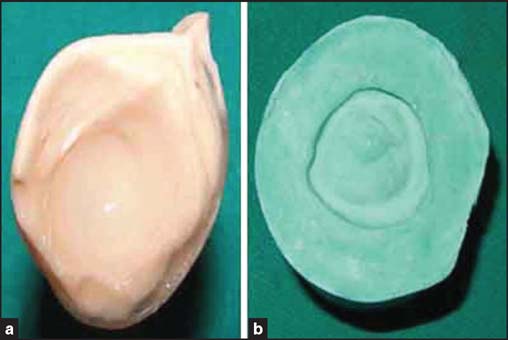
(a) Impression of the ocular defect. (b) Cast obtained after double-pour technique.

**Figure 3:**
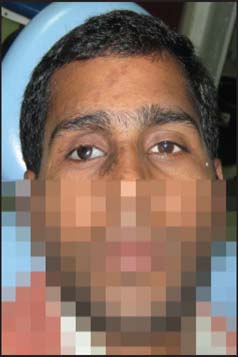
Final wax pattern try-in.

**Figure 4:**
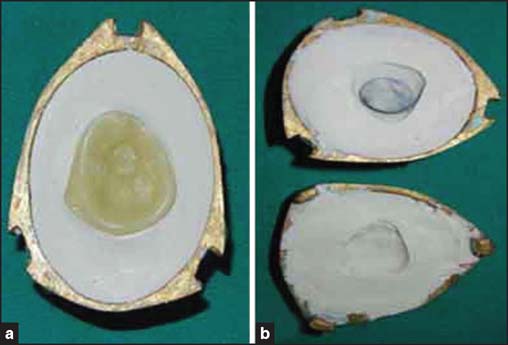
(a) Invested wax pattern. (b) Dewaxed mould.

A thin mixture of ophthalmic grade alginate (Opthalmic-moldite, Milton Roy Co., Sarasota, FL) was loaded into the syringe and injected into the socket to make a socket impression [Figure 2a] and the cast [Figure 2b] was poured in two parts. Then the wax pattern (Metrodent, Metro International Corp., Delhi, India) was made and adjusted in the patient’s eye socket. Finally the iris, matching in size and color of the contralateral eye, was obtained from a stock eye and positioned carefully on the wax pattern. A trial was done [Figure 3] and required corrections were made. This was invested, dewaxed [Figure 4a and b], and processed in tooth-colored acrylic (SC 10, Pyrax, Roorkee, India). Red silk fibers to mimic veins were placed in the acrylic dough followed by routine curing, finishing, and polishing. Finally, thin film of the sclera was removed and replaced by a clear film of transparent heat-cured PMMA (Trevalon, Dentsply India Pvt. Ltd., Gurgaon, India) to simulate corneal translucency

**Figure 5:**
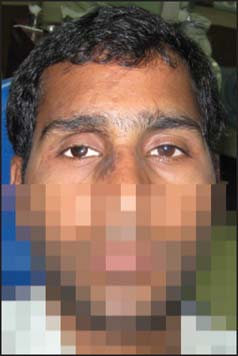
Postoperative photograph showing prosthesis in lieu of defective eye.

The final prosthesis was inserted in the socket [Figure 5] after disinfection and minor adjustments were made as per the patient’s comfort and aesthetics. Instructions regarding proper care and hygiene maintenance were given. The patient was advised to come for regular follow-ups at 1 month, 3 months, later followed by 6 monthly recall appointments. On 6 months recall appointments, further clinical and ophthalmic examination was performed and no complications with regard to health of underlying residual ocular tissues were found and there was no need of relining of the prosthesis.

## Discussion

Each individual copes in his unique way to the loss of eye. The rehabilitation of patients with phthisis bulbi presents a challenging clinical situation, as the patient has already been clouded by the sadness and psychological distress due to loss of vision and loss of facial aesthetics, as a result of failed ocular therapy. But this is not the end of the treatment and it should be emphasized that the cosmetic rehabilitation of these patients with ocular prosthesis is an integral part of their treatment, which fulfils aesthetic, psychological requirement of the patients and helps in their reintegration in the society.

Ocular prosthesis can be fitted over the phthisical eye, thereby eliminating the need for enucleation. Additional benefits of preserving the ocular remnants are reduced potential for development of anophthalmic socket syndrome and improved mobility of the ocular prosthesis. Usually, fabrication of custom ocular prosthesis involves complex and time-consuming painting procedures and is based purely on painting skills of the operator. The technique presented in this article modifies prefabricated eye prosthesis to a custom-made fit and aesthetics, which helps in overcoming the disadvantages of poor fit, inadequate movement and complex painting procedure and technique involved in making a custom-made ocular prosthesis. This technique is relatively easy to perform and time saving. Advantages include improved adaptation to underlying tissues, increased mobility of the prosthesis, improved facial contours, and enhanced aesthetics gained from control over the size of the iris and pupil and color of the iris and sclera.[9]

## Conclusion

Rehabilitation of patients with ocular trauma requires a multidisciplinary approach involving ophthalmologist, psychologist, and skilled maxillofacial prosthodontist. Custom-made ocular prosthesis fitted over the phthisical eye is to be a highly positive, logical, noninvasive, and beneficial approach to improve the cosmetic appearance and psychological well-being of the patients with ocular trauma.
